# Synovial membrane mesenchymal stem cells: past life, current situation, and application in bone and joint diseases

**DOI:** 10.1186/s13287-020-01885-3

**Published:** 2020-09-07

**Authors:** Na Li, Jinfang Gao, Liangyu Mi, Gailian Zhang, Liyun Zhang, Na Zhang, Rongxiu Huo, Junping Hu, Ke Xu

**Affiliations:** 1 Department of Rheumatology, Bethune Hospital Affiliated to Shanxi Medical University, Taiyuan, 030032 Shanxi China; 2 Department of Rheumatology, Shanxi Bethune Hospital, Taiyuan, 030032 Shanxi China

**Keywords:** Synovium mesenchymal stem cells, Immunophenotype, Cell subpopulation, Chondrogenic potential, Cartilage repair

## Abstract

Mesenchymal stem cells (MSCs) can be isolated from not only bone marrow, but also various adult mesenchymal tissues such as periosteum, skeletal muscle, and adipose tissue. MSCs from different tissue sources have different molecular phenotypes and differentiation potential. Synovial membrane (SM) is an important and highly specific component of synovial joints. Previous studies have suggested that the synovium is a structure with a few cell layers thick and consists mainly of fibroblast-like synoviocytes (FLS), which forms a layer that lining the synovial membrane on the joint cavity and synovial fluid through cell-cell contact. In recent years, studies have found that there are also mesenchymal stem cells in the synovium, and as an important part of the mesenchymal stem cell family, it has strong capabilities of cartilage forming and tissue repairing. This article reviews the sources, surface markers, subtypes, influencing factors, and applications in inflammatory joints of synovial membrane mesenchymal stem cells (SM-MSCs) in recent years, aiming to clarify the research status and existing problems of SM-MSCs.

## Background

Mesenchymal stem cells are derived from mesoderm mesenchymal stem cells with multi-directional differentiation potential. In 1968, Friedenstein et al. [1] found that a group of cells in the rat bone marrow can form fibroblast-like clones and be induced in vitro to differentiate into bone tissue, which also can reconstruct the blood microenvironment after being transplanted into the mouse. In 1991, the above-mentioned cells from the bone marrow were named MSCs for the first time [2]. Later, precursor cells of various tissues, such as bone marrow, muscle, fat, placenta, umbilical cord, and dental pulp, were collectively called MSCs [3]. According to the International Society for Cellular Therapy (ISCT), MSCs must meet the following characteristics when cultured in vitro: (1) MSCs should be mechanically adherent to and expand on plastic containers; (2) MSCs must be positive for expressing CD73, CD90, and CD105, but negative for the expression of CD45, CD31, CD34, CD14, CD11b, CD79α, CD19, and human leukocyte antigen (HLA)-DR surface marker molecules; and (3) MSCs must possess the ability to differentiate into osteoblasts, chondrocytes, and adipocytes under appropriate conditions [4]. Although the current research focuses on bone marrow-derived mesenchymal stem/stromal cells (BM-MSC), bone marrow is difficult to obtain and the numbers are also insufficient, which limits the widely clinic application of BM-MSCs. With the advancement of technology and the invention of sophisticated instruments, we have a deeper and more subtle understanding of disease research, which prompts us to be more willing to break the routine and explore new treatments and new sources of old diseases. Many studies have shown that the biological factors related to the immunoregulatory activity and specificity of MSC are largely dependent on the tissue from which the stem cells are derived. In addition to the widely studied common sources of MSCs (including BM MSCs, adipose tissue (AD)-MSC, and umbilical cord (UC)-MSC), research on MSCs from other sources should also be placed first in the future because these MSCs may have more unknown advantages and appropriate treatment effects for specific diseases. Therefore, many researchers focus on exploring new sources of MSCs.

In 2001, De Bari et al. [5] isolated MSCs from the synovium (SM) of human knee joints for the first time. Their study found that these cells had stem cell characteristics and the multidirectional differentiation potential in vitro, and then they named these cells synovial membrane mesenchymal stem cells (SM-MSCs). Many researchers are more interested in SM-MSCs because they have more accessible sources, high proliferation rate, low immunogenicity, and greater chondrogenic differentiation potential compared with MSCs from other sources. Based on these advantages, more and more researchers began to pay attention to the therapeutic potential and basic mechanism of SM-MSCs in the treatment of a series of diseases in vivo and in vitro. In this paper, we review the latest research on the tissue origin, biological characteristics, and different cell subsets of SM-MSCs and highlight the promising perspectives of SM MSCs in the repair of cartilage in bone and joint diseases.

## Origin of SM-MSCs

Anatomically, the synovial membrane (SM) can be divided into two layers: the lining layer and the sub-lining layer [6]. The lining layer communicates with the articular cavity, which is not supported by blood vessels and basement membrane. The sub-lining layer is a network of connective tissue composed of sparse cells and blood vessels [7]. The synovium with fibrous subsynovium is called fibrous synovium, and the synovium with adipose subsynovium is referred as adipose synovium. The MSCs derived from the adipose synovium usually refers to the MSCs derived from the infrapatellar fat pad [8].

At present, there is no clear conclusion about the origin of SM-MSCs. Initially, Nakagawa et al. [9] found that there are vascular channels connecting bone marrow to SM in collagen-induced arthritis rat (CIA) model, and Nagase et al. also found that the number of SM-MSCs is proportional to the degree of proliferation of synovial vessels. Hence, it is speculated that SM-MSCs may be migrated from bone marrow MSCs into synovial vessels, and then into synovial joint cavity through synovial vessels, however which is only found in animal experiments. Zvaifler et al. [10] identified the stem cells in the blood in 2000 and hold that SM-MSCs may come from progenitor cells that enter the joint through blood circulation. It was reported that [11] the fibroblast-like synoviocytes (FLSs) derived from synovium of patients with rheumatoid arthritis (RA) are SM-MSCs, and animal model studies of RA confirmed that MSCs derived from bone marrow (BM) accounts for 30% of SM-MSCs in RA, while only 1% of SM-MSCs in normal joints comes from bone marrow. Once synovitis occurs, a large quantity of BM-MSCs will be recruited into synovial joints. The persistent injury signal of local joint will induce BM-MSCs to flow into the damaged joint continuously, which results in its sharply increasing proportion in SM-MSCs. These studies support the hypothesis that SM-MSCs may come from other parts, but some studies have found that both SM-MSCs and synovial cells express lamellar bodies, so it is supported that SM-MSCs may come from synovial membrane tissue.

**Fig. 1:**
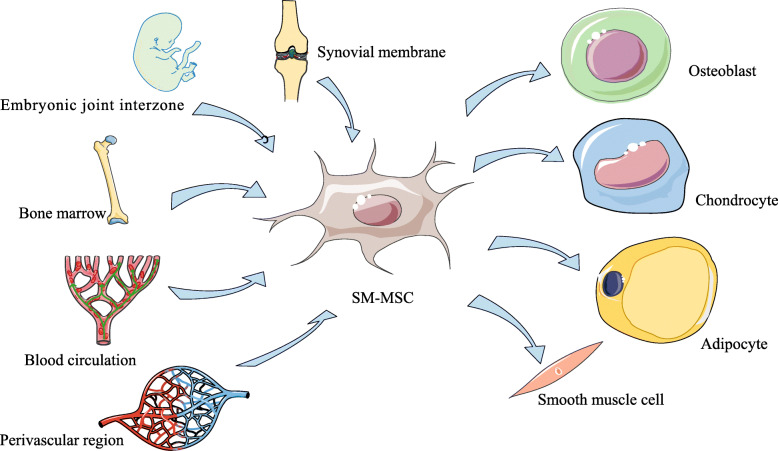
The possible origin and differentiation potential of synovial membrane mesenchymal stem cells (SM-MSCs)

In bone marrow, the perivascular region has been identified as the site of MSCs, and MSCs in the perivascular region is an important source of osteogenic and adipogenic lineage in adult bone marrow [12, 13]. Other studies have found that microvascular cells from the retina and the aorta also have the ability of multi-directional differentiation, which suggested SM-MSCs may also come from the perivascular region. Previous studies have found that vascular pericytes have the potential of osteogenesis and chondrogenesis, and compared with MSCs from other tissues, MSCs in the perivascular region is excellent in proliferation and cartilage differentiation. So, it is speculated that SM-MSCs may also come from stereotyped progenitor cells belonging to a specific lineage of vascular pericytes [14–16]. However, Roelofs et al. [17] found that the Gdf5^+^ cells in adult mice are MSCs with joint precursor activity. In order to support synovial lining layer cells’ proliferation and contribute to cartilage repair, the Gdf5+ cells will proliferate in the synovium in a Yes-related protein (Yap)-dependent manner after joint injury. Further studies confirmed that Gdf5 lineage cells also exist in adults, which come from the embryonic joint interzone and persist as SM-MSCs in the sub-lining layer of synovial membrane. After articular cartilage injury, Gdf5 lineage cells will proliferate and form new cartilage and, at the same time, be recruited into the Nes-GFP^bright^ population around synovial vessels, which co-expressed CD105 as well as SM22 and can further differentiate into myofibroblasts and smooth muscle cells and [18], finally, may support the angiogenesis. Roelofs’s data support that SM-MSCs come from the embryonic joint interzone, which is recruited around the blood vessels after cartilage injury rather than from perivascular area at first (Fig. 1).

## Biological characteristics of SM-MSCs

### Phenotype characteristics

Mochizuki et al. [8] showed that MSCs derived from human fibrous synovium and adipose synovium both express CD44 (hyaluronic acid receptor) and CD105, but with no or low expression of CD34 and CD45. The study also found that SM-MSCs have higher CD44 expression level in vitro than cells from other tissues [19–22]. CD44 plays an important role in the development of synovial joints disease and usually expressed on the surface or the internal area of the joints. When the joints are damaged and eroded, hyaluronic acid will bind to the CD44 on the surface and the synovium of the joints, which contributes to tissue separation and create a functional joint cavity. It is can concluded that CD44 may be the true symbol of SM-MSCs. It exists from early embryonic development to adulthood; however, like other MSCs markers, CD44 is not unique to SM-MSCs [23, 24].

Mochizuki et al. [8] found that the MSCs from fibrous synovium and adipose synovium compared with that derived from subcutaneous fat expressed higher level of STRO-1 and CD106, while lower CD10 level. STRO-1 was initially reported could be used to identify osteoblast precursor cells with colony-forming ability in the bone marrow [25], and recent studies have confirmed that it can be used in the phenotype identification of MSCs [26]. CD106 (VCAM-1) is a cell surface glycoprotein produced by endothelial cells activated by cytokines and is mainly expressed in the lining cells of synovial membrane tissue [27]. The positive rate of CD106 (VCAM-1) in cells derived from fibrous synovium and fat synovium was 5%, which was higher than that of cells derived from subcutaneous fat (1%); in the meantime, the positive rate of CD10 was 10%, which was lower than that derived from subcutaneous fat cells (40%). Colter et al. [28] confirmed that single-cell colonies derived from bone marrow-derived MSCs (BM-MSCs) contained three morphologically distinct cell types: large squamous cells, small spindle-shaped cells, and extremely small but rapidly dividing cells. It showed that compared with large squamous cells, small cells and extremely small cells have greater differentiation potential. They also found CD10 was just a negative marker for small cells and extremely small cells. Compared with MSCs from other tissues, SM-MSCs showed stronger proliferative capacity, chondrogenesis, and osteogenic potential, which may be the results of the existence of small cells and extremely small cells who express lower CD10.

**Fig. 2:**
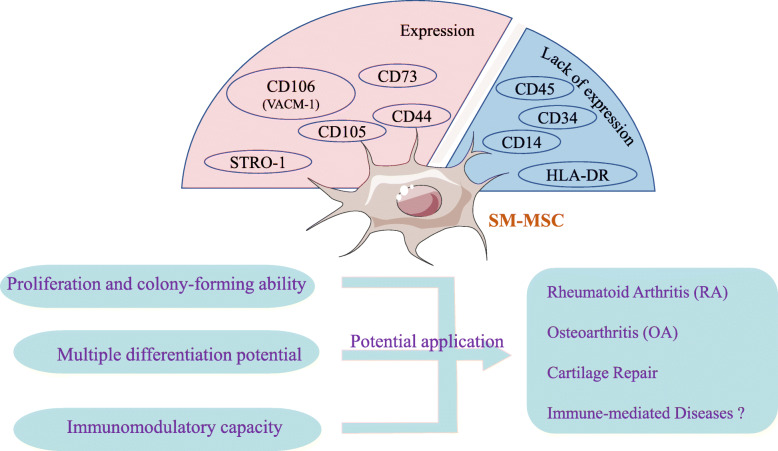
Possible clinical applications of synovial membrane mesenchymal stem cells (SM-MSCs)

In conclusion, the current research confirmed that SM-MSCs express CD44, CD105, CD73, CD166, CD90, CD106 (VCAM-1), and STRO-1 but not CD45, CD34, CD14, and HLA-DR [7, 8, 29](Fig. 2). However, it still failed to find surface markers specific for SM-MSCs, and the identification of SM-MSCs lacks a uniform standard recognized by the international community. Another study reported that there are different expressions of antigen markers when comparing the initially isolated and cultured MSCs population, which indicates that exposure to culture conditions will change the cell phenotype and some markers may be only the product of culture in vitro [30]. These findings suggest that it is still difficult to use only one marker to clearly characterize SM-MSCs. It may be more suitable for the verification of SM-MSCs to find the parallel expression or exclusion of several cell surface markers related to the pluripotency of mesenchymal stem cells differentiation.

### Proliferation and colony-forming ability

De Bari et al. [5] showed that human SM-MSCs can maintain their proliferative ability even after the 10th passage, and this proliferative ability does not seem to be related to donor age [31, 32]. Peiliang Fu et al. [33] found that the expansion rate of SM-MSCs in vitro experiments on rabbits is related to the inoculation density, low density is conducive to its rapid proliferation, while BM-MSCs in vitro are proliferated faster at high density. Interestingly, Sakaguchi et al. [34] also found similar results in the study of human SM-MSCs in vitro. Human SM-MSCs have higher self-renewal and expansion capacity than bone marrow-derived mesenchymal stem cells. The number of expanded human SM-MSCs inoculated at a lower density is greater than that of cells inoculated at a higher density. These studies suggest that cell senescence is affected by the number of cell passages, cell density, and cell population doubling time. Meanwhile, Mochizuki et al. [8] found that MSCs from different parts of the synovium of OA patients showed similar proliferative ability, suggesting that the proliferative ability of SM-MSCs has nothing to do with the location of their collection. It is worth noting that the number of nucleated cells per tissue weight in elderly OA patients was higher than in young patients with anterior cruciate ligament injury. The number of nucleated cells per tissue weight of fibrous synovium was higher than in adipose synovium, which suggested that the number of original SM-MSCs may be different. In addition, Mizuno et al. [14] confirmed that SM-MSCs in the perivascular area have higher proliferation potential than MSCs in other areas, while Mochizuki confirmed that MSCs derived from fibrous synovium and adipose synovium showed higher proliferation ability when compared with subcutaneous fat-derived MSCs [8].

Regarding the colony-forming ability of SM-MSCs, Nagase et al. found that it may be related to the number of α-smooth muscle actin positive blood vessels and the number of CD31^+^ endothelial cells [35]. The correlation between the colony-forming ability of SM-MSCs and the number of CD31^+^ endothelial cells is higher than that with the number of α-smooth muscle actin-positive blood vessels. CD31^+^ cells and α-smooth muscle actin-positive cells are located in different areas of the synovium, and there is also a correlation between the numbers of the two cells.

### Immunomodulatory capacity

Extensive research has been conducted on the effective immune regulation and anti-inflammatory ability of MSC. The MSCs exert their immunomodulatory properties by cell to cell contact and the secretion of immune regulatory molecules. It is well known that MSCs can inhibit the activation and proliferation of T and B lymphocytes, reduce the cytotoxicity of NK, block the proliferation and cytotoxicity of natural killer T cells, and weaken the maturation and antigen presentation ability of dendritic cells, as well as induce the phenotypic transformation of macrophages. MSC also has the function of inhibiting cell apoptosis and maintaining the viability of neutrophils. The known soluble factors involved in the immune regulation process of MSC are indoleamine 2,3-dioxygenase (IDO), nitric oxide, prostaglandin E2 (PGE2), hepatocyte growth factor (HGF), transforming growth factor (TGF)-b1, interleukin (IL)-6, and IL-10 (Fig. 1). However, the studies on the immunomodulatory capacity and anti-inflammatory effects of SM-MSCs are few.

Hagmann et al. [36] confirmed that SM-MSCs and BM-MSCs from the same OA patient have different immunomodulatory properties and surface markers. Co-culture of MSCs and allogeneic lymphocytes showed that SM-MSCs can significantly inhibit the proliferation of CD4^+^ T cells stimulated by CD3/CD28, while BM-MSCs from the same patient cannot. The researchers also observed that the number of T cells after co-culture was significantly reduced in late apoptosis and necrosis, suggesting that SM-MSCs have the ability to stabilize and protect T cell populations in vitro, which is consistent with the findings of Cuerquis [37]. In addition, compared with BM-MSC, the expression of HLA-DR on SM-MSC was significantly reduced, suggesting that its potential immunogenicity was reduced.

Recently, a group of researchers injected human SM-MSCs from OA patients into the CIA mice through the joint cavity. It was observed that TNF-α, IFN-γ, and IL-17A were reduced in mice, while IL-10 production increased. The number of Th1 and Th17 cells in the spleen of mice treated with SM-MSCs was decreased, while the number of Th2, Treg, PD-1^+^ CXCR5^+^ FoxP3^+^ follicular Treg cells, and IL-10-regulated B cells was increased. This study shows that human SM-MSCs derived from OA patients can exert their immunomodulatory properties in mice, which means the SM-MSCs can prevent the development of arthritis in mice and inhibit their abnormal immune responses and, finally, restore the peripheral tolerance of CIA mice by promoting the expansion of immune regulatory cells in CIA mouse. The study also showed a significant increase in the number of immature B cells in CIA mice treated with SM-MSCs, suggesting that SM-MSCs may inhibit B cell maturation and differentiation [38]. To our knowledge, this is the first study of using SM-MSC for CIA mouse. At the time of writing, a variety of clinical trials used MSC registered at ClinicalTrials.gov, most of which have been run for tissue repair and immune-mediated diseases such as GVHD, CD, MS, AA, and RA. However, there are no clinical trials using SM-MSC for immune diseases.

It is worth mentioning that the immune response ability of MSC will be affected by the level of inflammation. It is reported that MSCs are not constitutively immunosuppressive and they adopt immunosuppressive phenotype (MSC2) or pro-inflammatory phenotype (MSC1) according to the inflammatory environment in which they are located. In cases of high levels of pro-inflammatory cytokines or activation of the Toll-like receptor (TLR)-3, MSCs adopt immunosuppressive phenotype and inhibit the function of antigen-presenting cells (dendritic cells (DC), macrophages, B lymphocytes), T lymphocytes, NK cells, NKT cells, and neutrophils. When low levels of inflammatory cytokines are present or Toll-like receptor (TLR)-4 is activated, MSCs adopt pro-inflammatory phenotype, promote the activation of neutrophils and T cells, and enhance the immune response [39, 40]. In addition, MSCs can also act as antigen-presenting cells which result in pro-inflammatory reactions in some situations [41, 42]. This explains why MSCs do not exert its immunosuppressive effect but promote the pro-inflammatory process in some diseases involving inflammation. However, the more precise mechanism that triggers this contradiction effect needs further studies.

### Multiple differentiation potential

It has been reported that SM-MSCs can be expanded in vitro and have multi-lineage differentiation ability. SM-MSCs derived from fat synovium and fibrous synovium have similar characteristics in colony-forming efficiency and chondrogenic, osteogenic, and lipogenic abilities [21]. The cells harvested from the fibrous synovium and fatty synovium of young and old donors also showed similar self-renewal and differentiation ability, indicating that the differentiation ability of SM-MSCs was independent of donor age [8]. This study also found that the cartilage formation potential of SM-MSCs was similar in different parts of synovium of OA patients, which confirmed that the proliferation and differentiation potential of SM-MSCs were not affected by the harvested parts. The pellet wet weight is an important index to evaluate the chondrogenesis potential of a population of MSCs in vitro. Some researchers quantitatively compared the differentiation potential of MSCs from bone marrow, synovium, periosteum, adipose tissue, and muscle, which showed that SM-MSCs have the greatest differentiation potential among the mesenchymal tissue-derived cells examined [34, 43]. Compared with subcutaneous fat-derived MSCs, SM-MSCs have the same lipogenic ability but higher chondrogenic and osteogenic ability, colony-forming efficiency, and expansion ability [8].

Studies have reported that SM-MSCs have significant potential to regenerate articular cartilage and meniscus in various animal models [34, 44, 45]. Moreover, there is age-related decrease in the potential of cartilage formation and differentiation in periosteum [46] of rabbits and synovium [47] of organ culture in vitro, while no relevant report has been found in human body. In addition, it has been found that bone marrow-derived MSCs can form large myotubes [48], and De Bari [5] have shown that SM-MSCs can also form sporadic atypical myotubes, which proved that SM-MSCs have myogenic ability, which may be related to the cell culture environment.

In a word, SM-MSCs have superior multi-directional differentiation ability when compared with other mesenchymal stem cells (Fig. 1). We speculate that there may be two explanations for the differentiation difference of MSCs from different sources: on the one hand, the number of original MSCs is different in different region; on the other hand, the local tissue microenvironment may affect the differentiation bias of MSCs and induce them to differentiate to a specific lineage.

## Different cell subpopulations of SM-MSCs

As for the different subpopulations of synovial mesenchymal stem cells, there is no clear definition and classification, but many scholars at home and abroad have explored it for a long time. A team of researchers reported that the triple marker combination in CD9, CD44, CD54, CD90, and CD166 can be used to isolate MSCs from the synovium of patients with OA and confirmed that CD9/CD90/CD166 triple-positive cell subgroups have obvious chondrogenic and osteogenic differentiation ability [49]. But the researcher claimed that they were not sure whether such a combined marker could completely enrich the MSCs population. Another group of researchers successfully isolated a highly enriched population of MSCs from the bone marrow using a combination of cell surface markers LNGFR (CD271) and THY-1 (CD90) [50], confirming that the surface markers based on these two combinations can continuously and steadily concentrate MSCs. The same method was applied to the synovium and SM-MSCs were successfully isolated, and it confirmed that the CD9/CD271 double-positive subgroup in the synovium had a high chondrogenic potential [51]. But neither of these two studies pointed out the specific anatomical location of the subpopulation, nor did they compare the functional differences between the negatively labeled and positively labeled subpopulations.

Recently, Sivasubramaniyan et al. [30] reported that the combination of CD45, CD31, CD73, and CD90 can separate two different subpopulations of MSCs in the synovium of patients with OA. The subpopulation expressing the combination of CD31^−^CD45^−^CD73^+^CD90^+^ is located in the perivascular tissue in the sub-lining layer, while the subpopulation expressing CD31^−^CD45^+^CD73^+^CD90^−^ is located in the synovial lining layer. These two subpopulations of MSCs from different anatomical parts of the synovium will exhibit different cartilage differentiation potential when cultured in vitro and have clear cartilage repair ability. CD73^+^CD90^−^ cells can form cartilage only under the stimulation of TGFβ-1, while the CD73^+^CD90^+^ MSCs subgroup needs to be stimulated by BMP2 and TGFβ-1. The reason for the discrepancy is the addition of BMP2 can reverse the reduced chondrogenic capacity of CD73^+^CD90^+^ cells and increase the response based on TGF-β, suggesting that different cell subpopulations have different requirements for chondrogenic factors. Further research found that these subgroups of cells also exist in the synovium of healthy people, but the subgroups of MSCs derived from the synovium of healthy humans do not have different capacity of cartilage differentiation; synovial tissue which originates from OA patients may be responsible for this difference.

Mizuno et al. [14] selected the synovium from patients with OA and divided it into three regions on the histological level: surface, interstitial, and perivascular area. The surface area of the synovium is mainly composed of macrophages and fibroblasts. The interstitial area is defined as the subsynovial tissue except for the surrounding area of the blood vessel, which is composed of stromal cells with collagen fibers. The perivascular area mainly contains blood vessels and perivascular cells. They found MSCs are present in all three areas. Although the number of synovial cells extracted from the peritubular area is less than the number of synovial cells in the interstitial area, the perivascular MSCs have higher proliferation capacity and cartilage-forming potential whether expressed as a group or as a single cell. It is worth noting that perivascular MSCs also showed the highest COL10A1 mRNA expression ability, indicating that they have the potential to differentiate into hypertrophic chondrocytes.

Murata et al. [52] selected two parts for research: the paralabral synovium and the cotyloid fossa synovium, both of which come from the hip joint of femoroacetabular impingement syndrome patients. The anatomical location of the cotyloid fossa is defined in the lower part of the acetabulum, surrounded by the surface of the acetabular horseshoe-shaped crescent [53]. Murata’s research confirmed that SM-MSCs from the cotyloid fossa contained more adipose tissue than that in the paralabral region. The synovium in the cotyloid fossa area was pale yellow as visually observed while the paralabral synovium was whiter. The SM-MSCs in the cotyloid fossa are also relatively more advantageous in terms of cell yield, viability, proliferation and adipogenesis, cartilage formation, and osteogenic differentiation potential. Hatakeyama et al. [54] selected SM-MSCs from the paralabral region in the human knee joint and the hip joint to study, and then found the paralabral region of the knee joint contains more fat components than that of the hip joint, and SM-MSCs from the paralabral synovium in the knee joint have higher chondrogenic differentiation potential than do those from hip joint (Table 1).

**Table 1:** The detailed information of cell subpopulation in various research

Subpopulation	Outcome	Material sources	Donor age	Reference
CD9^+^CD90^+^CD166^+^	The potential for osteochondral differentiation	Patients with end-stage OA who underwent total knee joint replacement	55–82	Fickert et al. [49]
CD271^+^CD90^+^	The potential for chondrogenic differentiation	Patients with total knee arthroplasty surgery	N/A	Ogata et al. [51]
CD73^+^CD90^−^/CD73^+^CD90^+^	CD73^+^CD90^−^ cells can form cartilage only under the stimulation of TGFβ-1; the CD73^+^CD90^+^ MSCs subgroup needs to be stimulated by BMP2 and TGFβ-1	Patients with advanced clinical OA who underwent total knee replacement	50–80	Sivasubramaniyan et al. [30]
SM-MSCs in surface, interstitial and perivascular area	Higher proliferation and chondrogenic potential of MSCs in perivascular area	The knees of patients with osteoarthritis during total knee arthroplasty	59–85	Mizuno et al. [14]
SM-MSCs in the paralabral synovium and the cotyloid fossa synovium	MSCs from the cotyloid fossa synovium have higher proliferation and differentiation potential than do those from the paralabral synovium	Patients with femoroacetabular impingement syndrome but excluded osteoarthritis and inflammatory diseases	19–64	Murata et al. [52]
SM-MSCs in the cotyloid fossa area from the knee and hip	Adipogenesis and osteogenesis potentials of MSCs from the knees are superior to those of MSCs from the hips in the same donor	Patients underwent knee and hip arthroscopic surgeries	25–64	Hatakeyama et al. [54]

*N/A* not applicable

## Research progress of SM-MSCs in bone and joint diseases

### Study of SM-MSCs in osteoarthritis (OA)

Hip osteoarthritis (HO) is the most common joint disease among the old people. About 50% of the over 65-year-old people are affected, and the incidence of females is higher [55]. HO is the result of progressive degeneration of articular cartilage. It is known that degenerative changes of cartilage are related to mechanical stress of local tissues and inflammation-induced biochemical changes. It has been reported that MSCs play an important role in the pathogenesis of osteoarthritis, which have been identified in normal structures and diseased tissues [56, 57], but there is still little research on the role of SM-MSCs in the progression of HO disease. Turdean et al. [55] found CD105 and CD44 double-positive MSCs were present both in the lining and sub-lining layer of the hip joint, and it has been confirmed that the classic primary HO is mainly characterized by inflammatory infiltration around the blood vessel and simple synovium cell hyperplasia, while the rapidly destructive HO manifested as papillary synovial hyperplasia and the formation of germinal center in the sub-lining layer. The study also confirmed that the severity of rapidly destructive HO disease progression may be related to large-scale immune mobilization mediated by CD44/CD105 double-positive SM-MSCs.

Generally, CD44 and CD105 double-positive cells are rare on healthy synovium, but in experimental animal models of osteoarthritis (OA), the number of CD44/CD90 double-positive pluripotent stem cells with high proliferation capacity will increase significantly. OA is the most common chronic disease of synovial joints, characterized by the gradual loss of articular cartilage, which leads to pain and dysfunction. But OA is not a specific human disease; dogs can also develop OA spontaneously. CD44 is a single-pass transmembrane glycoprotein involved in cell-cell, cell-matrix adhesion, cell signaling, and many cell expressions [58]. It has been proved by research that the expression of CD44 is necessary to maintain the stability of articular cartilage [59] and CD44 is involved in the development of OA disease. The expression of CD44 will increase with the time of OA disease progression [60]. Study found that compared with the healthy control group, patients with primary knee OA had higher levels of CD44 expression. The expression intensity of CD44 in joints or synovium was significantly related to the severity of OA disease. CD44 may mediate the progression of OA disease in terms of inflammatory process and joint destruction [61].

Hermida-Gómez et al. [22] confirmed that the synovium of OA patients contains more CD44, CD90, and CD105 antigen-positive cells than normal joint synovium, and the number of cells expressing MSCs markers in OA synovium is twice that of normal synovium, which indicated that the number of SM-MSCs in OA is more than that of normal synovium, and these cells have been confirmed to have the ability to differentiate into chondrocytes in vitro. Further research found that only a part of the cells in the synovium-derived cell population are stem cells, and not all synovium cells have stem cell properties. The articular cartilage itself is avascular, so when the articular cartilage is damaged, it can only be repaired by itself or by surrounding tissues. Under normal circumstances, the body will initiate a spontaneous repair mechanism, that is, there will be a fibrous membrane tissue containing a small number of cell layers to spontaneously cover the damaged area of cartilage to resist cartilage damage, but the “spontaneous repair tissue” itself has no biomechanical effect, and eventually cartilage degradation process may occur. Hermida-Gómez found CD44 and CD90 antigen-positive cells are located in “spontaneous repair tissue,” but these cells did not express CD105 like other cells in the synovium. Considering that the ability of these cells to repair cartilage may be affected by the degradation process of cartilage, researchers speculated that the absence of CD105 may be necessary for repairing cartilage damage in OA. The MSCs with the potential for cartilage formation in the synovium may migrate into the damaged cartilage and thus participate in the active process of cartilage regeneration and repair.

In addition, studies have reported that the treatment of SM-MSCs for patients with OA is not a direct effect of a single injection but, in the case of maintaining the activity of live cells as well as the characteristics of the MSCs in the knee unchanged, inhibit the progression of OA disease through the secretion of nutritional factors [62].

### Study of SM-MSCs in rheumatoid arthritis (RA)

Rheumatoid arthritis (RA) is a systemic autoimmune disease characterized by persistent inflammation and extensive synovium hyperplasia and ultimately leads to articular cartilage and bone destruction [63]. Kohno et al. [20] found that the proliferation of SM-MSCs derived from RA and OA after 14 days in vitro culture was similar. The number of SM-MSCs that can be collected in patients with a history of methylprednisolone consumption is relatively low, and the number of SM-MSCs obtained from the OA patients who were injected with glucocorticoids in the knee before 1 week was also extremely low after 14 days of culture. All of these findings suggest that glucocorticoids may affect the number of SM-MSCs. The study also confirmed that the yield and surface markers of primary SM-MSCs in RA are similar to those of OA. There is no significant difference in the weight of cartilage sediments of SM-MSCs between RA and OA, indicating that the two have similar cartilage formation potential. This is consistent with previous results reported by Skasska [64] and Koizumi [65], but Jones et al. [66] reported that the cartilage formation potential of SM-MSCs derived from RA is higher than that of SM-MSCs derived from OA. Jones et al. confirmed that the expression level of CD44 on RA SM-MSCs was higher than that of OA. The expression of CD44 may be negatively correlated with the synovial inflammation in RA and positively correlated with the chondrogenesis potential of MSCs. In view of previous research having reported that the cartilage differentiation ability of BM-MSCs will be inhibited by inflammatory cytokines such as interleukin (IL)-1β, tumor necrosis factor alpha (TNFα), and IL-17, it is speculated that the difference of chondrogenesis potential of SM-MSCs derived from RA in these different research groups may be due to the different inflammatory levels of the selected donors in each experimental study.

The imbalance of the proportion of effector cells and regulatory cells caused by the loss of autoimmune tolerance plays an important role in the pathogenesis of RA [67, 68]. Studies have shown that SM-MSCs may be involved in the regulation of immune homeostasis in healthy joints, and the failure of this immune regulation is the basis of the development of RA. SM-MSCs have the ability to inhibit the proliferation of T cells in vitro, and it has been confirmed that RA SM-MSCs have the same ability to inhibit the proliferation of T cells in vitro as the SM-MSCs from healthy donors. Djouad and Zhang et al. [69, 70] confirmed that the addition of TNF-α can reverse the inhibition of MSCs on T cell proliferation under inflammatory conditions, which is conducive to the stem cell therapy of RA. However, Gazdic et al. [39, 40] reported that the immune response of MSCs will be affected by the level of inflammation. Huang et al. [71] reported that in the inflammatory environment of RA patients, there are high levels of TLR4 in the synovium. Abnormal activation of TLR4 will promote MSCs to adopt pro-inflammatory phenotype. It is speculated that SM-MSCs may act as antigen-presenting cells, leading to the activation and proliferation of T cells in the inflammatory environment of RA, and then cooperate with the abnormal immune system to promote the continuation of inflammation [72]. Arthritis will not only lead to joint damage, but also inhibit the joint regeneration potential of SM-MSCs and affect its immune regulation ability. There are few studies on RA SM-MSCs at present; moreover, most of these studies are limited in vitro. There are many studies supported that the joint inflammation of RA will affect the function of local MSCs. Any therapeutic intervention measures for the formation of new cartilage in RA induced by SM-MSCs should include the effective inhibition of local inflammation. Considering that in vitro culture of MSCs may reduce the environmental impact of original joint inflammation on MSCs function, the future research should focus on uncultured (in vivo) RA SM-MSCs.

### Study of SM-MSCs in cartilage repair

Kurth et al. [73] confirmed that the adult synovium is a site of functional MSCs that contributes to cartilage repair after joint injury. It is said that the synovium has the same embryonic origin as articular cartilage [74] and SM-MSCs can migrate to the site of cartilage injury and play a role in repairing cartilage defects [30]. Many researchers have conducted explorations on SM-MSCs in cartilage repair in animal models. Koga et al. [44] implanted MSCs isolated from adult rabbit bone marrow, synovium, adipose tissue, and muscle into the full-thickness cartilage defect of the rabbit knee; it confirmed that these MSCs can be differentiated into corresponding cartilage tissues in the rabbit, and the cartilage matrix formed by synovial and bone marrow-derived MSCs is higher than that of fat and muscle. Nagase et al. used SM-MSCs to repair cartilage defects in rabbit knee joints, whose results showed that the implanted MSCs can differentiate into cartilage tissue suitable for the local microenvironment, which also confirmed the cartilage formation potential of SM-MSCs.

Given that the knee joints of pigs are similar in size and cartilage-specific properties to those of humans, many researchers have chosen pigs as animal models to study the effectiveness of SM-MSCs transplantation. Nakamura et al. [75] injected fluorescently labeled allogeneic SM-MSCs suspension into the osteochondral defect in pigs; 4 weeks later, they found the osteochondral defect was filled with cartilage matrix. Kondo et al. [76] selected autologous SM-MSCs aggregates to investigate whether SM-MSCs could successfully repair the cartilage defects in microminipigs in the medial femoral condyle and femoral groove. Their results showed that the transplantation of SM-MSC aggregates promoted the regeneration of the articular cartilage in the medial femoral condyle of microminipig but not in the femoral groove, suggesting that the site of cartilage defect may also affect the cartilage repair ability of stem cells. At the same time, the researchers also found that the regenerated tissue appeared to be less than fully mature, which may be related to the shorter follow-up time. Moreover, it is worth noting that the stem cell transplantation in this study was performed immediately after the formation of the cartilage defect, which is different from the clinical situation. In addition to the direct transplantation of stem cells, a Japanese research team has generated a scaffold-free tissue-engineered construct (TEC) which comprises autologous SM-MSCs and extracellular matrix synthesized by the stem cells for cartilage repair. The researchers also select porcine as animal models for preclinical research to repair cartilage lesions. Finally, biomechanical analysis demonstrated that the cartilage tissue generated by TEC transplantation showed similar characteristics to normal cartilage tissue. This animal experiment proved that TEC can successfully repair damaged cartilage [77–80].

Based on these preclinical studies, in 2015, the Japanese research group stepped forward to a clinical research [81]. In this study, they implanted TECs into the cartilage defects (1.5–3.0 cm^2^) of 5 patients without fixation and then evaluated the safety and effectiveness of using TEC for cartilage repair within 2 years. The results showed that no adverse events were recorded in the follow-up observation, and the self-assessment and clinical scores of pain, symptoms, activities of daily living, sports activities, and quality of life were significantly improved. This study once again proved the potential of SM-MSCs in the repair of cartilage damage.

Surprisingly, during the process of collecting SM-MSCs to construct TEC, researchers found that the SM-MSCs of a male who was one of the subjects in this clinical trial and received a week of high-dose steroid therapy for Bell's palsy failed to generate a functional TEC. Further studies confirmed that the patient’s stem cell function was temporarily impaired because of the use of steroid hormones. The effect of steroids in vivo is similar to the direct effect of the drug on MSCs [82]. This finding indicates that we should pay close attention to the time of SM-MSC collection for stem cell therapies, as well as assess the medication situation of the donors and recipients in order to maximize the function of stem cells.

Sekiya et al. [83] conducted a clinical study involving 10 patients with a symptomatic single cartilage lesion in the femoral condyle. First, the SM-MSCs suspension was placed on the cartilage defect with a syringe under arthroscopic control. After a 3-year follow-up study, they found that the transplantation of SM-MSC significantly improved the MRI features, histologic features, and clinical evaluation scores in patients with cartilage defects in the knee. In conclusion, all these evidence support the potential application of SM-MSCs in the cartilage repair. Recently, another study by Sekiya also demonstrated the potential of SM-MSCs in repairing meniscus damage [84]. However, all these clinical studies have several common limitations, namely the small sample size, short follow-up time, and no control group. We look forward to new breakthroughs in these areas in future studies.

## Problems and outlook

In conclusion, SM-MSCs, as a member of stem cell family, are easy to obtain, have obvious plasticity and strong proliferation ability in vitro, and have a wide application prospect in bone and joint diseases. Although there have been many studies on the proliferation, multi-differentiation potential, and immune characteristics of SM-MSCs in vitro, most of the related studies are limited to in vitro and animal experiments. In the future, we need to improve the experimental research in vivo.

Current research has shown that not all cells in the synovium have stem cell properties. Whether the inflammatory activity in the synovium will change the composition of synovial cells and lead to changes in the functional characteristics of the stem cell population requires further research and exploration. Pathological research is particularly important to clarify the mechanism of action of bone and joint diseases such as OA and RA and the characteristics of SM-SMCs.

The functional characteristics of different subsets of SM-MSCs are quite different, so the next step is to accurately identify the cell surface markers or transcription factors to distinguish the differentiation potential between different subsets, which is particularly important to clarify the biological function and application of SM-MSCs. In view of the low immunogenicity of SM-MSCs and the ability to inhibit the proliferation of T/B cells, we propose that SM-MSCs also have broad application potential in immune-mediated diseases. We believe that SM-MSCs play an important role in the occurrence and development of bone and joint diseases such as RA and OA. The strong chondrogenic differentiation potential of SM-MSCs in vitro provides new ideas and methods for regenerative medicine and stem cell therapy of bone and joint diseases (Fig. 2). However, obviously lots of effective preclinical trials still need to run before SM-MSCs can be used for clinical treatment in future.

## Conclusion

In summary, although further studies are needed, SM-MSC-based treatment has great potential for cartilage and tissue repair in various diseases. As SM-MSCs are a type of adult stem cells having a myriad of therapeutic properties, further elucidation of its mechanism of action and differences in biological characteristics between normal person and patients are necessary for future clinical applications.

## Data Availability

Please contact the corresponding author for data requests.

## Abbreviations

MSCs: Mesenchymal stem cells
SM: Synovial membrane
BM: Bone marrow
AD: Adipose tissue
UC: Umbilical cord
SM-MSCs: Synovial membrane mesenchymal stem cells
BM-MSCs: Bone marrow mesenchymal stem/stromal cells
ISCT: International Society for Cellular Therapy
HLA: Human leukocyte antigen
FLS: Fibroblast-like synoviocytes
CIA: Collagen-induced arthritis rat
RA: Rheumatoid arthritis
HO: Hip osteoarthritis
OA: Osteoarthritis
TNF: Tumor necrosis factor
TEC: Tissue-engineered construct
